# Health-related quality of life from 20 to 32 years of age in very low birth weight individuals: a longitudinal study

**DOI:** 10.1186/s12955-022-02044-3

**Published:** 2022-09-14

**Authors:** Elias Kjølseth Berdal, Arnt Erik Karlsen Wollum, Ingrid Marie Husby Hollund, Johanne Marie Iversen, Eero Kajantie, Kari Anne I. Evensen

**Affiliations:** 1 Faculty of Medicine and Health Sciences, Norwegian University of Science and Technology, Trondheim, Norway; 2 Department of Clinical and Molecular Medicine, Norwegian University of Science and Technology, Trondheim, Norway; 3 Department of Physical Medicine and Rehabilitation, St. Olavs Hospital, Trondheim University Hospital, Trondheim, Norway; 4 Department of Internal Medicine, Nordland Hospital Trust, Bodø, Norway; 5 Department of Clinical Medicine, UiT Arctic University of Norway, Tromsø, Norway; 6 Finnish Institute for Health and Welfare, Public Health Promotion Unit, Helsinki, Oulu, Finland; 7 PEDEGO Research Unit, MRC Oulu, Oulu University Hospital and University of Oulu, Oulu, Finland; 8 Children’s Hospital, Helsinki University Hospital and University of Helsinki, Helsinki, Finland; 9 Unit for Physiotherapy Services, Trondheim Municipality, Trondheim, Norway; 10 Children’s Clinic, St. Olavs Hospital, Trondheim University Hospital, Trondheim, Norway; 11 Department of Rehabilitation Science and Health Technology, Oslo Metropolitan University, Oslo, Norway

**Keywords:** Health-related quality of life, Longitudinal, Long-term outcome, Preterm, Self-perceived health status, Short Form 36 Health Survey, SF-36, Very low birth weight, Young adulthood

## Abstract

**Background:**

Preterm birth with very low birth weight (VLBW, birth weight < 1500 g) is associated with health problems later in life. How VLBW individuals perceive their physical and mental health-related quality of life (HRQoL) is important to understand their putative burden of disease. Previous studies have shown mixed results, and longitudinal studies into adulthood have been requested. This study aimed to investigate differences in HRQoL between preterm VLBW and term born individuals at 32 years of age, and to study changes in HRQoL from 20 to 32 years.

**Methods:**

In a geographically based longitudinal study, 45 VLBW and 68 term born control participants completed the Short Form 36 Health Survey (SF-36) at 32 years of age. Data from three previous timepoints was also available (20, 23 and 28 years of age). The SF-36 yields eight domain scores as well as a physical and a mental component summary. Between-group differences in these variables were investigated. We also performed subgroup analyses excluding individuals with disabilities, i.e., cerebral palsy and/or low estimated intelligence quotient.

**Results:**

At 32 years of age, the physical component summary was 5.1 points lower (95% confidence interval (CI): 8.6 to 1.6), and the mental component summary 4.1 points lower (95% CI: 8.4 to − 0.3) in the VLBW group compared with the control group. For both physical and mental component summaries there was an overall decline in HRQoL from 20 to 32 years of age in the VLBW group. When we excluded individuals with disabilities (n = 10), group differences in domain scores at 32 years were reduced, but physical functioning, bodily pain, general health, and role-emotional scores remained lower in the VLBW subgroup without disabilities compared with the control group.

**Conclusion:**

We found that VLBW individuals reported lower HRQoL than term born controls at 32 years of age, and that HRQoL declined in the VLBW group from 20 to 32 years of age. This was in part, but not exclusively explained by VLBW individuals with disabilities.

**Supplementary Information:**

The online version contains supplementary material available at 10.1186/s12955-022-02044-3.

## Background

Neonatology is a rapidly evolving field in medicine, and the survival rate of preterm born individuals with low birth weight has drastically improved over the last decades [1, 2]. Modern medicine has improved the survival rate into adulthood for preterm infants in industrialised countries to 95% [3], resulting in an increasing population of adults born preterm. As the preterm infants are exposed to extrauterine environment and immaturity-related illness [3–10], the holistic consequences of this challenge for the individual as well as for the society is important to investigate. Studies show that individuals born preterm with very low birth weight (VLBW, birth weight < 1500 g) have a higher risk of chronic conditions [3], e.g. poorer lung function [4], motor problems [5], cognitive impairment, relational disorders and autism traits [6], psychiatric disorders [7] and more internalising behaviour than their term born peers [8–10].

Subjective valuation of quality of life has become increasingly recognised as an important outcome both in medical care and clinical research [11]. Thus, to understand the burden of disease among individuals born preterm with VLBW and its implications on life in a more comprehensive manner, measuring health-related quality of life (HRQoL) may be useful. This can provide important complementary information to the traditional functional outcomes from the perspective of the individual [11]. Furthermore, longitudinal assessment of quality of life using repeated measures to permit observations of changes over time are encouraged [12]. However, the definition and conceptual framework of HRQoL is not clear [11]. The World Health Organization has defined health as “a state of complete physical, mental and social well-being and not merely the absence of disease or infirmity” [13] and quality of life as “the individual’s perception of their position in life in the context of culture and value systems in which they live and in relation to their goals, expectations, standards and concerns [14]. This was based on three basic characteristics of quality of life; subjectivity, multi-dimensionality, and positive and negative dimensions [14]. Thus, the concept of HRQoL refers to a broad approach of looking at a person’s health, including the psychological, physiological and social impact on the total well-being of an individual [15].

According to a systematic review from 2020 there is no conclusive evidence that HRQoL differs between VLBW adolescents and their term born peers [16]. However, some studies were inconclusive, and the authors requested longitudinal studies investigating the transitional phase into adulthood and how this affects the VLBW individuals in their daily lives [16]. This important and unique era in life between childhood and adulthood, is challenging in many ways. Studies have shown that for preterm born individuals, impairments may become more evident when leaving the parental home and establishing a family, building a career, and having social relations [17–19]. The NTNU Low Birth Weight in a Lifetime Perspective study (NTNU LBW Life) has examined HRQoL as well as other outcomes in a population of preterm VLBW and term born controls, throughout their twenties. Based on previous findings, we have speculated that the transitional phase into adulthood is an especially difficult time and can increase differences between the two populations [20].

The primary aim of this study was to investigate possible differences in HRQoL between preterm born VLBW and term born control individuals at 32 years of age. Secondly, we wanted to determine the development of HRQoL from 20 to 32 years of age in the VLBW population. We hypothesised that the demands following the transition into adulthood, may negatively affect the self-perceived quality of life.

## Methods

### Study design

This is a geographically based, longitudinal study of VLBW individuals and term born controls. The VLBW individuals were born between 1986 and 1988 and admitted to the neonatal intensive care unit at St. Olavs Hospital (Trondheim University Hospital, Norway), formerly known as Trondheim Regional Hospital. The control participants were born at term in the same period and geographical area. The participants have been invited to follow-up visits at several timepoints from childhood to adulthood. In this study, we used data from the Short Form 36 Health Survey (SF-36) at four timepoints in adulthood (20, 23, 28 and 32 years of age). The 32-year data collection was carried out between 2019 and 2021.

### VLBW group

**Fig. 1:**
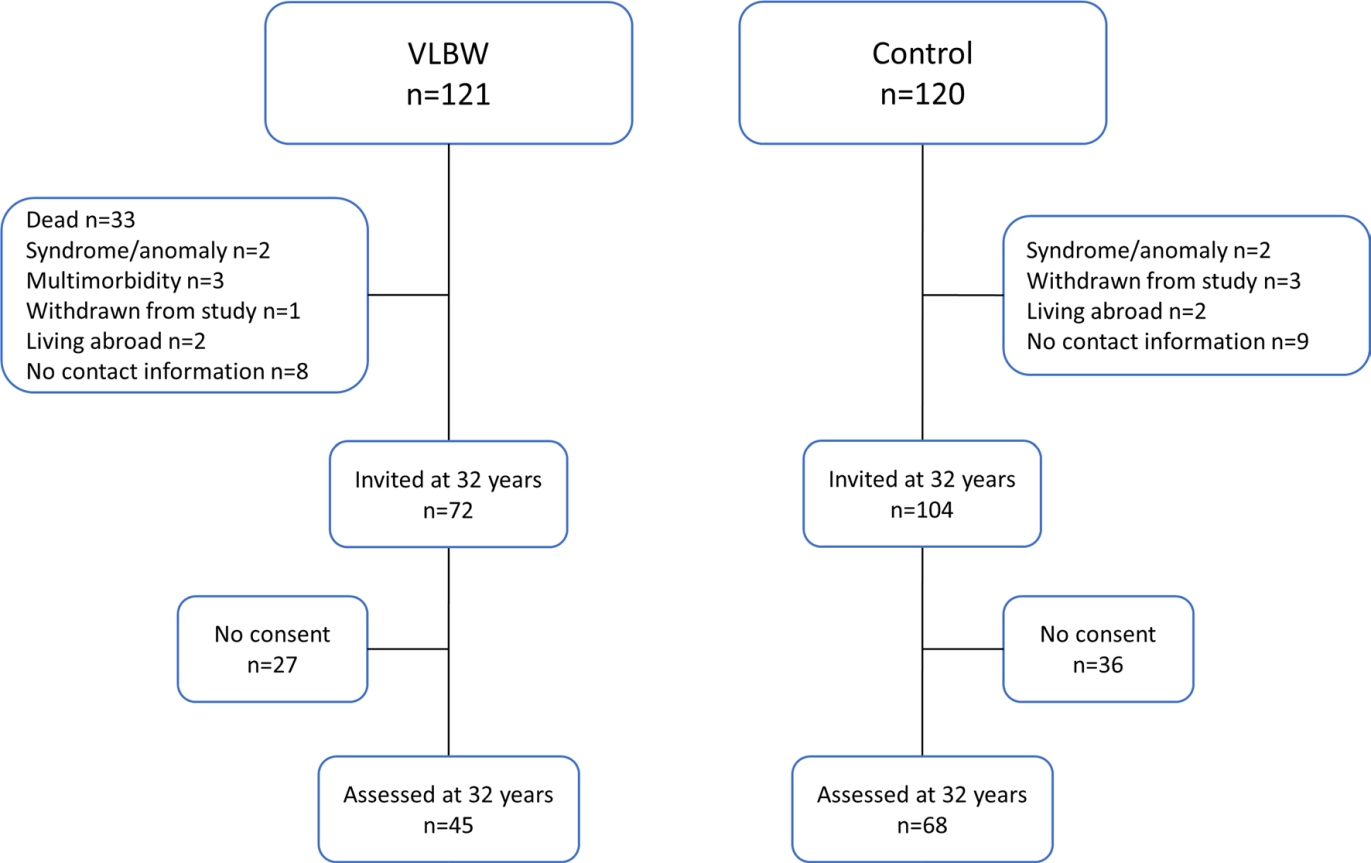
Flow of participants. VLBW =  very low birth weight

The VLBW group originally included 121 individuals with a birth weight ≤ 1500 g (Fig. 1). Of these, 33 died in the neonatal period, five were excluded due to syndromes/multimorbidity, and one withdrew from the study. Of 82 eligible candidates, eight where without contact information and two were living abroad. Thus, 72 VLBW adults were invited to the study, of these 45 consented to participation. At 20, 23 and 28 years of age, 52, 35 and 51 VLBW individuals participated, respectively. Flow of participants for these follow-up timepoints are published previously [20–22]. Altogether, 68 VLBW individuals had available data on SF-36 at one or more timepoints.

### Control group

The control group comprised 120 individuals from a multicentre study born at term with birth weight ≥ 10th percentile for gestational age, corrected for sex and parity [23]. A random 10% of pregnant women was selected for follow up, whereof the ones residing in the Trondheim area were included in the NTNU LBW Life study. At 32 years of age, two were not eligible due to congenital malformations, three had withdrawn from the study, nine were without contact information and two were living abroad. Hence, 104 were invited to participate, whereof 68 consented to participation. At 20, 23 and 28 years of age, 74, 37 and 86 controls participated, respectively. Altogether, 100 control individuals had available data on SF-36 at one or more timepoints.

### Non-participants

There were no differences in infants’ perinatal data, parental socioeconomic status (SES), proportion of cerebral palsy (CP), low estimated intelligence quotient (IQ) or previous SF-36 results at 20, 23 and 28 years of age between participants and those who did not consent to participation at 32 years of age in either group. However, in the VLBW group, those who did not consent were born to younger mothers (mean difference: − 3.0 years [95% CI: − 5.2 to − 0.7], *p* = 0.005), had lower birth weight (mean difference: − 129 g [95% CI: − 239 to − 18], *p* = 0.023) and gestational age (mean difference: − 1.4 weeks [95% CI: − 2.7 to − 0.1], *p* = 0.030), and were more likely to be men (77.8% vs. 42.2%, *p* = 0.003) compared with participants.

### Background variables

Data on birth weight, gestational age and sex was recorded at birth. Information on complications in the perinatal period were retrieved from hospital records. Parental SES were recorded at the 14 and 19 year follow-up visits, based on a combination of education and occupation of both parents according to Hollingshead Two-Factor Index of Social Positioning [24]. At 14 years of age, cerebral palsy (CP) was diagnosed by project paediatricians and IQ was estimated by using two subscales of Wechsler Intelligence Scale for Children - third edition; Vocabulary and Block Design [25]. Low estimated IQ more than two standard deviations (SD) below the mean in the control group and/or presence of CP was defined as having a disability.

### Outcome measure

The SF-36 is a self-report questionnaire consisting of 36 items, which includes eight different domains: Physical functioning, role limitations due to physical problems (role-physical), bodily pain, general health, vitality, social functioning, role limitations due to emotional problems (role-emotional) and mental health [26]. Physical functioning (10 items) is related to performance of activities, such as running, lifting, domestic life, walking distances and activities of daily living, while role limitations due to physical problems (four items) includes having to cut down time, accomplish less, being limited or having difficulty carrying out daily activities [27]. Bodily pain (two items) includes pain magnitude and interference of pain in daily activities. General health (five items) comprises a rating of perceived health as a rating of whether one gets more easily sick or is as healthy as others, expect health will get worse or have excellent health. Vitality (four items) includes being full of pep and energy, worn out or tired. Social functioning (two items) is related to influence of health on extent and time being social, while role limitations due to emotional problems (three items) includes having to cut down time, accomplish less or perform activities less careful. Mental health (five items) includes being nervous, down, peaceful, sad or happy. Each domain is represented with a score from 0 to 100, where higher scores indicate better HRQoL. The two domains of role-physical and role-emotional has dichotomised response choices, while the other domains have a Likert-type response option with three to six choices. The recall period is four weeks, expect for physical functioning and general health, which address current status. Three of the domains (physical functioning, role-physical and bodily pain) contribute mainly to a physical component summary, while three other domains (social functioning, role-emotional and mental health) contribute mainly to a mental component summary. The domains of general health, vitality and social functioning have noteworthy correlations with both component summaries [27]. The two composite summaries of mental and physical HRQoL are calculated from a T-score with a population average of 50 points. Factor-analytic studies have confirmed physical and mental health factors that account for 80–85% of the reliable variance in the eight scales in the US general population, among patients and in general populations in Sweden and the United Kingdom [26]. The reliability of the eight scales and two summary measures has been estimated using both internal consistency and test–retest methods. In more than 25 studies, published reliability statistics have been at least 0.70; most have exceeded 0.80, and the reliability estimates for physical and mental summary measures usually exceed 0.90 [26]. The Norwegian version of SF-36 has been evaluated in a Norwegian registry population of patients with rheumatoid arthritis between 20 and 79 years of age (n = 1552), and found to have acceptable reliability and validity [28]. Internal consistency estimates for the scales (Cronbach’s alphas) ranged from 0.74 for the domain of general health to 0.91 for physical functioning [28].

### Statistical analyses

The data were analysed in SPSS, version 27. Statistical significance was set at two-sided *p*-values below 0.05. Group differences in the SF-36 and background characteristics were analysed with chi-square statistics for categorical data, Student *t*-test for independent samples for continuous and normally distributed data, and Mann–Whitney U test for ordinal data or continuous data with a non-normal distribution. Group differences in SF-36 domains and component summaries were analysed using linear regression. Estimated changes in domains and component summaries from 20 to 32 years were analysed using linear mixed models. SF-36 scores were entered separately as dependent variables, time and group and their interaction (time x group) as fixed factors, sex as fixed factor, and participant as random factor. Both analyses require the residuals to be normally distributed. Normality of residuals was judged by visual inspection of Q–Q plots. Due to some deviations from normality, we used bootstrapping with B = 2000 bootstrap samples and bias corrected and accelerated (BCa) method. Ninety-five percent confidence intervals (CI) are reported where relevant. We also performed a subgroup analysis excluding individuals with CP and/or low estimated IQ.

### Ethical considerations

This study was conducted in accordance with the Helsinki Declaration, and the study was approved by the Regional Committee for Medical and Health Research Ethics in Central Norway (23879). Written informed consent was given by all participants.

## Results

### Clinical characteristics

Table 1 shows clinical characteristics of the two study groups. Maternal age, parental SES and sex were comparable between the two groups.

**Table 1:** Clinical characteristics of very low birth weight participants and term born controls

	VLBW			Control		
	n	Mean	(SD)	n	Mean	(SD)
Birth weight (g)	45	1223.6	(218.5)	68	3695.3	(459.2)
Gestational age (weeks)	45	29.3	(2.3)	68	39.8	(1.2)
Birth head circumference (cm)	38	27.3	(2.2)	64	35.4	(1.2)
Apgar score after 1 min	44	6.8	(2.2)	63	8.9	(0.5)
Apgar score after 5 min	44	8.6	(1.6)	64	9.8	(1.2)
Maternal age at birth (years)	45	29.1	(5.2)	66	30.7	(4.3)
Parental SES	41	3.3	(1.2)	57	3.7	(1.1)

		n	(%)		n	(%)
Female	45	26	(57.8)	68	39	(57.4)
Cerebral palsy	45	2	(4.4)	68	0	
Low estimated IQ	45	8	(18)	61	0	

Low estimated IQ = estimated intelligence quotient < 2SD of the mean in the control group, SD = standard deviation, SES = socioeconomic status, VLBW = very low birth weight

### Health-related quality of life at 32 years of age

Table 2 shows the SF-36 scores at 32 years of age in the VLBW group with and without disabilities compared with the control group. The VLBW group had lower scores, indicating a lower HRQoL, in the domains of physical functioning, role-physical, general health, role-emotional, and mental health. The mean differences in domain scores between the two groups ranged from 6.4 points (95% CI: − 0.5 to 13.2) in mental health, to 21.0 points (95% CI: 8.0– to 34.0) in the role-physical domain. The physical and mental component summaries showed a mean difference of 5.1 (95% CI: 1.6 to 8.6) and 4.1 points (95% CI: − 0.3 to 8.4), respectively.

**Table 2:** Health-related quality of life in participants born VLBW and controls at 32 years

	VLBW (n = 45)				VLBW without disabilities^a,b^ (n = 35)				Control (n = 68)	
	Mean	(SD)	Mean difference versus control (95% CI)		Mean	(SD)	Mean difference versus control (95% CI)		Mean	(SD)
*Domains*										
Physical functioning	87.1	(19.2)	9.2	(3.2 to 15.2)	90.3	(16.7)	5.8	(0.8 to 10.7)	96.3	(7.2)
Role-physical	71.7	(40.5)	21.0	(8.0 to 34.0)	78.6	(37.4)	13.6	(− 0.2 to 27.5)	92.6	(19.8)
Bodily pain	68.2	(30.6)	10.0	(− 0.4 to 20.4)	73.1	(28.7)	4.4	(1.0 to 15.5)	78.2	(20.5)
General health	64.3	(25.5)	14.0	(5.2 to 22.8)	65.4	(27.0)	11.4	(1.0 to 21.7)	78.3	(18.5)
Vitality	48.6	(21.6)	6.5	(− 1.0 to 14.1)	50.4	(22.6)	5.2	(− 3.1 to 13.5)	55.1	(18.4)
Social functioning	81.7	(26.5)	8.2	(− 1.0 to 17.4)	83.6	(27.2)	6.4	(− 4.2 to 16.9)	89.9	(19.7)
Role-emotional	71.1	(39.9)	21.0	(7.9 to 34.2)	77.1	(36.8)	16.3	(2.5 to 30.1)	92.2	(23.1)
Mental health	73.1	(20.0)	6.4	(− 0.5 to 13.2)	75.2	(20.3)	4.9	(− 2.2 to 12.0)	79.4	(14.1)
*Component summaries*										
Physical component	50.0	(10.8)	5.1	(1.6 to 8.6)	51.5	(10.8)	3.2	(− 0.7 to 7.2)	55.2	(5.5)
Mental component	46.6	(12.4)	4.1	(− 0.3 to 8.4)	47.6	(13.0)	3.5	(− 1.6 to 8.5)	50.6	(9.6)

CI = Confidence interval, SD = standard deviation, VLBW = very low birth weight

^a^Without cerebral palsy and/or estimated intelligence quotient < 2SD of the mean in the control group

^b^Compared with 61 controls due to missing estimated intelligence quotient for seven control participants

When we excluded ten VLBW individuals with CP and/or low estimated IQ, group differences in domain scores were reduced, but physical functioning, bodily pain, general health, and role-emotional scores remained lower compared with term born controls. The differences in physical and mental component summaries were 3.2 (95% CI: − 0.7 to 7.2) and 3.5 points (95% CI: − 1.6 to 8.5), respectively.

### Longitudinal changes in health-related quality of life from 20 to 32 years of age

**Fig. 2:**
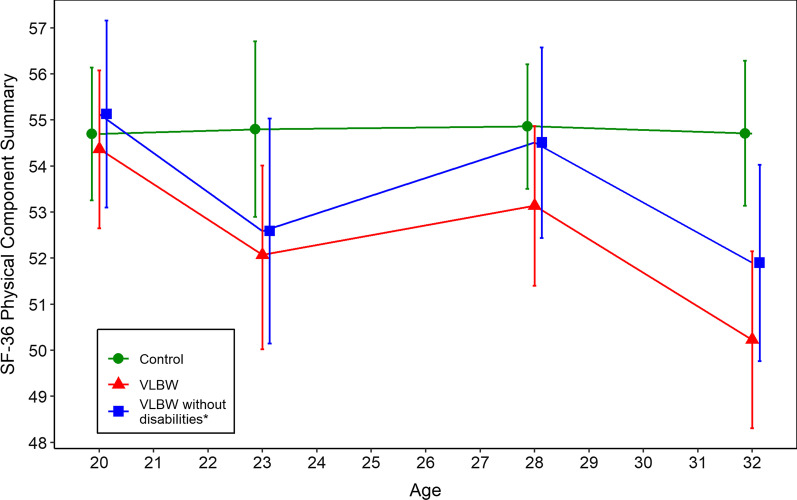
Physical component summary of Short Form 36 Health Survey from 20 to 32 years. T-scores are given with 95% confidence intervals. A higher score indicates better physical health-related quality of life. SF-36 = Short Form 36 Health Survey, VLBW = very low birth weight. *Without cerebral palsy and/or estimated intelligence quotient below two standard deviations of the mean in the control group

**Fig. 3:**
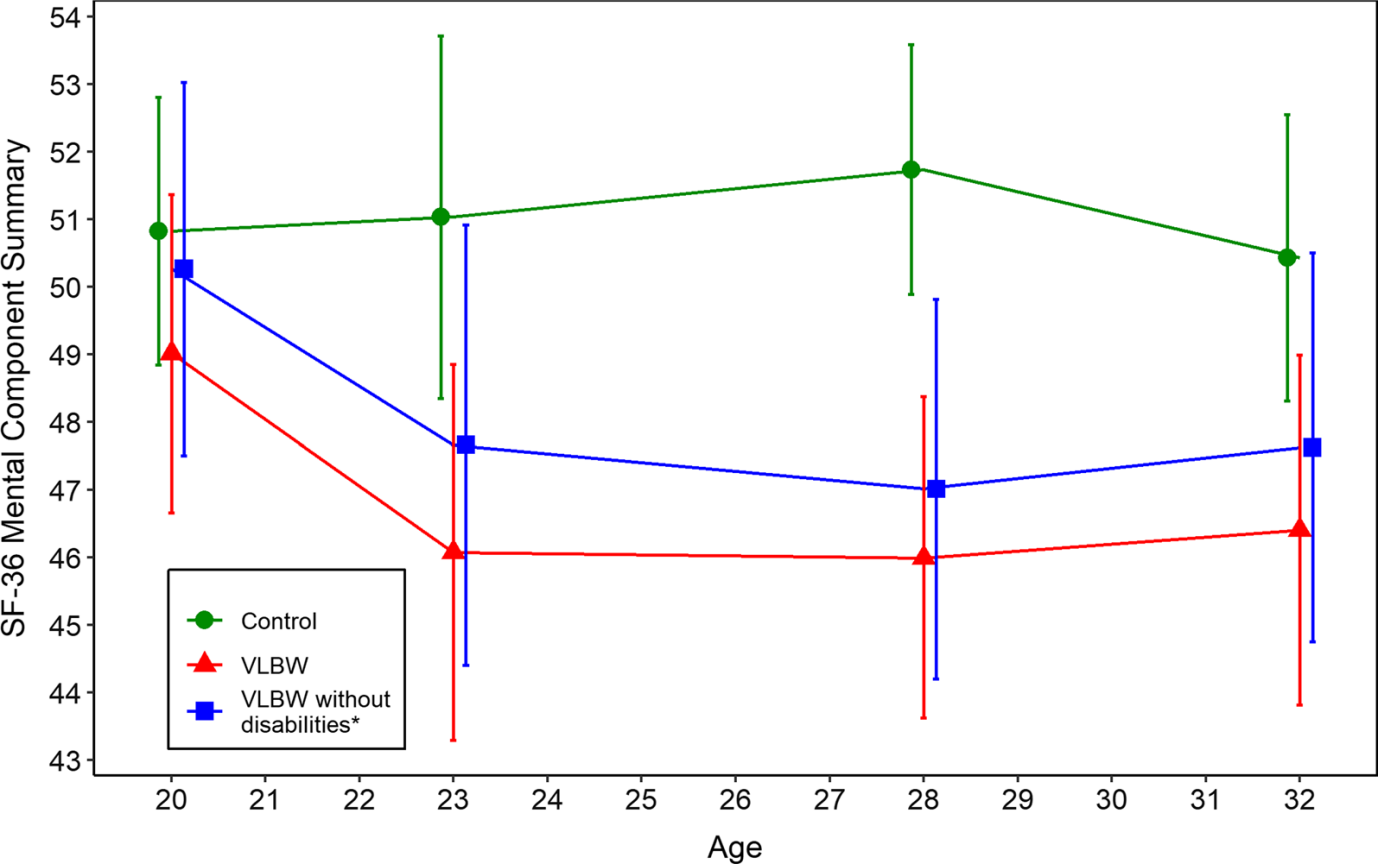
Mental component summary of Short Form 36 Health Survey from 20 to 32 years. T-scores are given with 95% confidence intervals. A higher score indicates better mental health-related quality of life. SF-36 = Short Form 36 Health Survey, VLBW = very low birth weight. *Without cerebral palsy and/or estimated intelligence quotient below two standard deviations of the mean in the control group

Group differences of the SF-36 at 20, 23 and 28 years of age are shown in Additional file 1: Table S1; Additional file 2: Table S2 and Additional file 3: Table S3. Table 3, Figs. 2 and 3 illustrate changes in domains and component summaries across all four timepoints in the VLBW and control group. The domains of general health and role-emotional differed over time between the two groups, as did changes in the physical and mental component summaries (*p*-value for interaction time x group = 0.024 and *p* = 0.056, respectively). From 20 to 23 years, there was a decline in both component summaries in the VLBW group, thereafter the mental component summary stabilised. The physical component summary was stable from 23 to 28 years but showed a further decline of − 2.9 points (95% CI: − 5.3 to − 0.3) from 28 to 32 years. Both component summaries were stable over time in the control group.

**Table 3:** Estimated changes in health-related quality of life from 20 to 32 years

	VLBW (n = 68)			Control (n = 100)			*p*-value time x group
	20–23 years	23–28 years	28–32 years	20–23 years	23–28 years	28 to 32 years	
	B (95% CI)	B (95% CI)	B (95% CI)	B (95% CI)	B (95% CI)	B (95% CI)	
*Domains*							
Physical functioning	− 1.8 (− 5.6 to 1.5)	2.8 (− 1.8 to 6.9)	− 4.9 (− 9.6 to 0.5)	0.5 (− 2.0 to 3.1)	0.5 (− 2.2 to 3.0)	− 0.7 (− 2.0 to 0.2)	0.308
Role physical	− 8.5 (− 17.5 to − 1.4)	− 0.4 (− 11.4 to 12.2)	− 6.5 (− 17.4 to 4.1)	4.5 (− 1.0 to 10.3)	− 6.0 (− 11.1 to − 1.1)	2.4 (− 2.8 to 7.5)	0.061
Bodily pain	− 11.5 (− 19.7 to − 3.0)	5.4 (− 2.7 to 13.6)	− 2.6 (− 10.3 to 5.9)	0.9 (− 4.1 to 6.0)	− 0.3 (− 5.6 to 5.1)	− 2.2 (− 5.7 to 1.0)	0.102
General health	− 10.1 (− 14.5 to − 6.3)	4.5 (− 2.0 to 11.2)	− 9.5 (− 15.2 to − 4.1)	− 4.7 (− 10.1 to 0.2)	8.9 (3.7 to 15.0)	− 4.8 (− 7.4 to − 3.3)	< 0.001
Vitality	− 4.1 (− 8.8 to 0.4)	0.6 (− 4.0 to 4.9)	0.3 (− 4.9 to 6.0)	− 1.7 (− 6.6 to 3.1)	2.4 (− 2.6 to 8.2)	− 2.1 (− 5.3 to 0.6)	0.640
Social functioning	− 4.8 (− 10.6 to 0.9)	− 1.8 (− 7.9 to 3.8)	− 1.7 (− 8.4 to 5.7)	1.1 (− 2.2 to 4.0)	0.5 (− 3.5 to 5.5)	− 3.7 (− 6.8 to − 0.5)	0.118
Role emotional	− 12.6 (− 21.6 to − 4.0)	0.6 (− 10.6 to 11.2)	− 6.5 (− 15.2 to 2.8)	6.0 (− 1.3 to 12.2)	− 2.7 (− 9.1 to 4.2)	− 1.7 (− 5.7 to 2.5)	0.001
Mental health	− 5.2 (− 9.5 to − 1.3)	1.9 (− 2.8 to 6.7)	2.3 (− 2.6 to 7.9)	− 1.1 (− 4.9 to 2.0)	3.6 (− 0.8 to 8.7)	− 1.8 (− 4.2 to 0.4)	0.226
*Component summaries*							
Physical component	− 2.6 (− 4.6 to − 0.8)	1.6 (− 0.7 to 3.7)	− 2.9 (− 5.3 to − 0.3)	0.1 (− 1.6 to 1.7)	− 0.1 (− 1.5 to 1.6)	− 0.1 (− 1.1 to 0.6)	0.024
Mental component	− 3.2 (− 5.7 to − 0.9)	− 0.1 (− 3.0 to 2.7)	0.5 (− 2.6 to 3.6)	0.1 (− 2.3 to 2.1)	1.2 (− 1.3 to 4.0)	− 1.4 (− 2.9 to 0.2)	0.056

CI = Confidence interval, SD = standard deviation, VLBW = very low birth weight

When we excluded individuals with disabilities, the trajectories for the mental and physical component summaries in the VLBW group showed a similar pattern of decline from 20 to 32 years of age (Figs. 2 and 3), however, the group differences in change over time were no longer statistically significant (*p*-value for interaction time x group = 0.082 for the physical and *p* = 0.184 for the mental component summary). Still, the group differences in change over time were significant for the domains of general health and role-emotional (*p*-value for interaction time x group = 0.001 and *p* = 0.002) with similar trajectories as for the whole VLBW group (Additional file 4: Table S4).

## Discussion

### Main findings

We found that 32-year-olds born with VLBW report lower HRQoL than their peers born at term. This was seen in general health, physical functioning, role limitations due to physical and emotional problems, mental health as well as in the physical component summary. Furthermore, the trajectories of physical and mental health from 20 to 32 years of age showed an overall decline for VLBW adults while remaining stable in the control group. Both component summaries declined from 20 to 23 years of age in the VLBW group. While the physical component summary declined further from 28 to 32 years of age, the mental component summary stabilised at 23 years of age. When we excluded VLBW participants with CP and/or low estimated IQ, the HRQoL trajectories showed the same pattern of declining HRQoL, however differences in HRQoL compared with the control group were reduced.

### Strengths and limitations

Strengths of this study includes the longitudinal design with measurements of HRQoL at several timepoints in the same population. However, loss to follow-up is inevitable in long-term follow-up studies [29] and may threaten the validity. There were few differences in background characteristics, but VLBW individuals who did not consent to participation at 32 years of age were born to younger mothers, had lower birth weight and gestational age, and were more likely to be men than participants. This could impact the outcome, making our findings more conservative, which is often the case with attrition bias [30]. The small sample size in our study may have affected the statistical power to detect differences, making the study vulnerable to type II errors, especially when excluding participants with disabilities. With low statistical power, it may be more relevant to focus on mean group differences instead of p-values. Due to the relatively small sample size, stratified analyses by sex were not performed. However, we adjusted for sex in the longitudinal mixed model analysis.

The SF-36 is a validated questionnaire which provides a broad comprehension of quality of life, acknowledging the three basic characteristics of quality of life; subjectivity, multi-dimensionality, and positive and negative dimensions [14]. Although the Norwegian translation was evaluated in patients with rheumatoid arthritis, the reliability and validity of the Norwegian translation used in this study are comparable with estimates from other countries [23]. Self-report questionnaires have both flaws and advantages. It is susceptible to social desirability bias, but less than interview based methods [31]. Furthermore, cognitive ability may affect one’s self-perception and ability to understand each question. Still, the self-report method is considered the best way to investigate HRQoL [16]. In longitudinal studies, it may be relevant to consider a response shift effect, i.e., whether the respondent’s view of their health-related quality of life may change over time due to changes of internal standards, values or the conceptualisation of the construct of interest [32]. However, as a response shift is typically occurring when individuals are adjusting or accommodating to an illness leading to a better evaluation of their life situation with time [32], it can be argued that those who have been born preterm with VLBW have adapted to their situation long before entering adulthood and that this would therefore not affect our results. If anything, it would imply that our results are conservative estimates of HRQoL in the VLBW group at 32 years of age.

We defined disability as having CP or estimated IQ more than two standard deviations lower than the mean in the control group. However, there is no consensus as to what definition of disability one should use, this makes it hard to compare our results of the subgroup analyses with other studies.

### Consistency with previous research

The most recent systematic review in this field reported inconclusive findings of HRQoL in VLBW and extremely low birth weight (ELBW, birth weight < 1000 g) populations [16]. Van der Pal et al. [16] included 18 studies, whereof 11 did not find a difference, three studies were inconclusive and four found a significant difference in HRQoL. As stated by the authors, it is a difficult task to compare HRQoL in preterm studies because of different outcome measures, sources of information, age at follow-up and weight limits for inclusion of participants [16]. Most of the studies reviewed included participants in the first half of their twenties. However, two studies reported a lower HRQoL in VLBW/ELBW individuals aged 26 [33] and 29–36 years [34]. Unfortunately, these two studies did not use the SF-36 and are therefore not directly comparable to our study. Among the seven studies using the SF-36, Båtsvik et al. [35] found lower scores for three of the eight domains in their ELBW population compared with term born controls at 24 years of age. However, they did not report the component summaries. Poole et al. [36] found no difference between a Canadian ELBW group and controls in any of the domains at 23 years of age, even though their inclusion criterion of ELBW individuals could imply larger group differences than in our study. However, they did not include individuals with neurosensory impairments such as CP, deafness, blindness, or intellectual disability. They also stated a high likelihood of attrition bias, which could underestimate their findings [30]. Natalucci et al. [27] used the SF-36 in a Swiss ELBW population at 23 years of age. They found that the mental component summary was lower, and the physical component summary was higher compared with community norms from a German and French population in 1997 and 2001. This may not be directly comparable to our study which included a control group, since it is shown that HRQoL scores provided by patients tends to be higher than those of community norms [37]. Three other studies found no difference between VLBW individuals and controls at the age of 19–22 years [38–40]. These findings are partly consistent with the findings of no difference in HRQoL at 20 years of age in our study.

We are aware of only one other study examining long-term trajectories of HRQoL up to the thirties in a VLBW/ELBW population. The Canadian McMaster Ontario cohort of ELBW individuals studied HRQoL from 12 to 36 years of age [34]. They found a decreasing HRQoL with age in the ELBW group, similar to the results of our study. These findings are in contrast to a systematic review by Zwicker and Harris [15] on HRQoL in VLBW, ELBW, and/or preterm born individuals from preschool to adult age. They found diminishing differences in HRQoL with age and hypothesised that the difference in HRQoL would fade completely into adulthood. However, they stated that the diminishing HRQoL were possibly reflected by issues related to parent-proxy vs. self-report, and the adaption of an individual’s challenges over time [15]. The present study showed that at 20 years of age there was little to no difference in HRQoL between the two groups, however, group differences increased after the age of 20 years.

When we excluded individuals with disabilities at 32 years of age, group differences in the physical and mental component summaries were reduced and no longer significant compared with the control group, suggesting that the VLBW group is diverse and has an uneven burden of disease. However, the domain scores of physical functioning, bodily pain, general health and role-emotional were still lower. At 24 years of age, Båtsvik et al. [35] found that three of the domains (i.e., social functioning, role-emotional and mental health) that comprise the mental aspect of HRQoL differed between the ELBW and the control group when excluding individuals with disabilities. This may be concurrent with our finding that the group difference in the mental component summary, even though not statistically significant, was less affected than the physical component summary when we excluded participants with disabilities. However, our finding of a poorer physical functioning, also when we excluded individuals with disabilities, contrasts the finding of Båtsvik et al. [35]. Their definition of disability included mainly physical disabilities, while we also included low estimated IQ, which may explain the discrepancy. The overall decline in HRQoL from 20 to 32 years of age, also for the VLBW individuals without disabilities, is concurrent with the McMaster Ontario cohort [34]. However, both our study and the McMaster study found that when excluding the most severely affected subgroup of VLBW individuals, the difference in HRQoL compared with the term born control group was reduced.

### Underlying mechanisms

Mechanisms that may explain our findings of poorer physical functioning and general health, also seen in VLBW individuals without disabilities, could be related to pulmonary function, muscular fitness, and motor functioning. A large individual participant meta-analysis has documented reduced expiratory airflow of the lungs [4]. A Finnish birth cohort study showed that young adults born early preterm (< 34 weeks of gestation) had lower muscular fitness than controls and perceived themselves as less fit than controls [41], and several reviews have shown poorer motor skills in children, adolescents and young adults born very preterm or VLBW [5, 42, 43]. Both VLBW individuals with and without disabilities reported poorer mental and emotional functioning, consistent with “the preterm behavioural phenotype” of inattention, anxiety, and social difficulties [6]. Two comprehensive meta-analyses have shown long-term mental health consequences of being born preterm with VLBW into adulthood, especially internalising problems [10], as well as anxiety, mood disorders and attention-deficit hyperactivity disorder amongst other psychiatric diagnoses [44]. Furthermore, their lower educational achievements may pose additional challenges compared to their peers entering adulthood [35, 45]. Thus, our findings seem reasonable considering what is already known about the outcomes of being born preterm with VLBW.

### Clinical implications

Preterm birth is influencing many aspects of future health. It is recommended that quality of life measures is integrated in studies on long-term outcomes of children with disabilities or chronic diseases [46]. The VLBW population may be considered as such a group, as it has an increased risk of chronic disorders and health problems that vary both in magnitude and diversity [3, 5–10, 47, 48]. The increased risk of developmental problems early in life may manifest in poorer adult physical health and earlier aging [48, 49]. The decline in physical HRQoL between the two time points at 28 and 32 years of age for the VLBW group could indicate that the increasing age already at a rather early phase of adulthood is more abrasive for the VLBW group compared with the rest of the population. However, HRQoL is a complicated outcome measure, which may be affected by cognitive function, social desirability bias, resilience, and adaptability to one’s situation [50], amongst many other factors.

This study contributes to the awareness and understanding of how being born with VLBW may impact an increasing group of people in our society. Our results could imply that health professionals should improve efforts to enhance physical, social and emotional functioning, and thereby quality of life, in preterm children [15] and that preterm birth should be a part of a comprehensive medical history of adult patients. Longitudinal HRQoL studies are a scarcity and are needed also in older populations to see how the changing HRQoL of VLBW individuals evolve into their late adulthood.

## Conclusion

In this study, we found that VLBW individuals reported lower HRQoL than term born controls at 32 years of age, measured by the SF-36. This was seen in general health as well as in physical, mental and emotional domains. Furthermore, HRQoL declined in the VLBW group from 20 to 32 years of age, indicating that increasing age rather early in adulthood is more abrasive for VLBW individuals compared with the rest of the population. Our results bring attention to the importance of assessing health and functioning from the individual’s perspective.

## Supplementary Information

**Additional file 1: Table S1** Health-related quality of life in participants born VLBW and controls at 20 years

**Additional file 2: Table S2** Health-related quality of life in participants born VLBW and controls at 23 years

**Additional file 3: Table S3** Health-related quality of life in participants born VLBW and controls at 28 years

**Additional file 4: Table S4** Estimated changes in health-related quality of life from 20 to 32 years in participants without disabilities

## Data Availability

The datasets generated and/or analysed during the current study are not publicly available because permission has not been applied for from neither the participants nor the Ethical Committee but are available from the corresponding author on reasonable request.

## Abbreviations

BCa: Bias-corrected and accelerated bootstrap
CI: Confidence interval
CP: Cerebral palsy
ELBW: Extremely low birth weight
HRQoL: Health-related quality of life
IQ: Intellectual quotient
NTNU LBW Life: NTNU Low birth weight in a lifetime perspective
SD: Standard deviation
SF-36: Short Form 36 Health Survey
VLBW: Very low birth weight
