# Supplementary material for: Health-related quality of life from 20 to 32 years of age in very low birth weight individuals: a longitudinal study
Source: Health Qual Life Outcomes. 2022 Sep 14;20:136. doi: 10.1186/s12955-022-02044-3 (PMC9476299; doi:10.1186/s12955-022-02044-3)
Supplement: Supplementary file 1 — Additional file 1: Table S1 Health-related quality of life in participants born VLBW and controls at 20 years [file 12955_2022_2044_MOESM1_ESM.docx]

**Table S1** Health-related quality of life in participants born VLBW and controls at 20 years

|  | **VLBW**  (n = 52) | | | **VLBW without disabilities^b^**  (n = 40) | | | **Control**  (n = 74) | |
| --- | --- | --- | --- | --- | --- | --- | --- | --- |
|  | Mean | (SD) | p-value vs. control | Mean | (SD) | p-value vs. control | Mean | (SD) |
| Domains |  |  |  |  |  |  |  |  |
| Physical functioning | 91.1 | (18.7) | 0.114 | 95.8 | (10.7) | 0.944 | 95.6 | (10.0) |
| Role-physical | 87.0 | (23.5) | 0.308 | 88.8 | (24.6) | 0.586 | 91.2 | (22.1) |
| Bodily pain | 80.3 | (21.7) | 0.981 | 81.9 | (21.6) | 0.693 | 80.2 | (22.5) |
| General health^a^ | 79.5 | (17.1) | 0.820 | 81.9 | (15.0) | 0.339 | 78.7 | (19.8) |
| Vitality^a^ | 51.0 | (18.8) | 0.092 | 52.9 | (18.1) | 0.279 | 56.2 | (14.2) |
| Social functioning | 89.7 | (13.9) | 0.209 | 90.9 | (12.7) | 0.480 | 92.7 | (13.1) |
| Role-emotional^a^ | 89.1 | (22.6) | 0.733 | 92.5 | (17.7) | 0.648 | 90.5 | (23.7) |
| Mental health^a^ | 74.0 | (15.1) | 0.034 | 76.8 | (13.0) | 0.329 | 79.2 | (11.9) |
| Component summaries |  |  |  |  |  |  |  |  |
| Physical component^a^ | 54.5 | (5.9) | 0.690 | 55.5 | (5.8) | 0.648 | 54.9 | (6.1) |
| Mental component^a^ | 48.9 | (7.9) | 0.155 | 49.9 | (7.0) | 0.485 | 51.0 | (7.7) |

IQ = intelligence quotient, SD = standard deviation, VLBW = very low birth weight.

^a^Data missing for one control participant.

^b^Without cerebral palsy and/or estimated intelligence quotient <2SD of the mean in the control group.
