# Supplementary material for: Health-related quality of life from 20 to 32 years of age in very low birth weight individuals: a longitudinal study
Source: Health Qual Life Outcomes. 2022 Sep 14;20:136. doi: 10.1186/s12955-022-02044-3 (PMC9476299; doi:10.1186/s12955-022-02044-3)
Supplement: Supplementary file 2 — Additional file 2: Table S2 Health-related quality of life in participants born VLBW and controls at 23 years [file 12955_2022_2044_MOESM2_ESM.docx]

**Table S2** Health-related quality of life in participants born VLBW and controls at 23 years

|  | **VLBW**  (n = 35) | | | **VLBW without disabilities^b^**  (n = 24) | | | **Control**  (n = 37) | |
| --- | --- | --- | --- | --- | --- | --- | --- | --- |
|  | Mean | (SD) | p-value vs. control | Mean | (SD) | p-value vs. control | Mean | (SD) |
| Domains |  |  |  |  |  |  |  |  |
| Physical functioning | 90.4 | (13.6) | 0.032 | 94.2 | (8.9) | 0.380 | 96.1 | (6.8) |
| Role-physical | 80.0 | (30.8) | 0.009 | 87.5 | (23.3) | 0.154 | 95.4 | (13.0) |
| Bodily pain | 68.7 | (28.4) | 0.022 | 77.0 | (23.9) | 0.439 | 82.1 | (18.8) |
| General health | 72.0 | (19.0) | 0.543 | 72.0 | (16.9) | 0.641 | 74.7 | (18.4) |
| Vitality^a^ | 49.0 | (14.0) | 0.053 | 50.2 | (13.6) | 0.175 | 55.4 | (13.5) |
| Social functioning | 86.1 | (16.5) | 0.023 | 90.1 | (12.8) | 0.170 | 94.3 | (13.0) |
| Role-emotional | 78.1 | (33.3) | 0.010 | 87.5 | (25.7) | 0.211 | 95.5 | (19.5) |
| Mental health^a^ | 70.7 | (16.8) | 0.031 | 74.5 | (13.7) | 0.201 | 78.7 | (13.4) |
| Component summaries |  |  |  |  |  |  |  |  |
| Physical component^a^ | 52.3 | (6.0) | 0.028 | 53.8 | (5.4) | 0.381 | 55.2 | (4.9) |
| Mental component^a^ | 46.7 | (9.0) | 0.020 | 48.7 | (7.6) | 0.154 | 51.3 | (6.8) |

IQ = intelligence quotient, SD = standard deviation, VLBW = very low birth weight.

^a^Data missing for one control participant.

^b^Without cerebral palsy and/or estimated intelligence quotient <2SD of the mean in the control group.
