# Supplementary material for: Health-related quality of life from 20 to 32 years of age in very low birth weight individuals: a longitudinal study
Source: Health Qual Life Outcomes. 2022 Sep 14;20:136. doi: 10.1186/s12955-022-02044-3 (PMC9476299; doi:10.1186/s12955-022-02044-3)
Supplement: Supplementary file 3 — Additional file 3: Table S3 Health-related quality of life in participants born VLBW and controls at 28 years [file 12955_2022_2044_MOESM3_ESM.docx]

**Table S3** Health-related quality of life in participants born VLBW and controls at 28 years

|  | **VLBW**  (n=51) | | | **VLBW without disabilities^a^**  (n=38) | | | **Control**  (n=86) | |
| --- | --- | --- | --- | --- | --- | --- | --- | --- |
|  | Mean | (SD) | p-value vs. control | Mean | (SD) | p-value vs. control^b^ | Mean | (SD) |
| Domains |  |  |  |  |  |  |  |  |
| Physical functioning | 92.3 | (11.6) | 0.038 | 95.3 | (9.0) | 0.337 | 96.1 | (7.6) |
| Role-physical | 77.5 | (34.7) | 0.031 | 83.6 | (30.9) | 0.256 | 89.8 | (26.4) |
| Bodily pain | 72.4 | (25.7) | 0.08 | 77.1 | (23.2) | 0.298 | 79.6 | (21.8) |
| General health | 74.3 | (23.3) | 0.046 | 77.2 | (23.2) | 0.180 | 81.7 | (16.0) |
| Vitality | 48.5 | (19.3) | 0.016 | 50.2 | (20.4) | 0.109 | 56.5 | (17.9) |
| Social functioning | 83.8 | (22.3) | 0.01 | 86.8 | (20.5) | 0.088 | 93.2 | (15.2) |
| Role-emotional | 77.8 | (33.8) | 0.006 | 82.5 | (29.8) | 0.055 | 92.6 | (21.3) |
| Mental health | 71.1 | (20.5) | 0.004 | 73.5 | (20.5) | 0.042 | 80.8 | (13.4) |
| Component summaries |  |  |  |  |  |  |  |  |
| Physical component | 53.1 | (8.2) | 0.201 | 54.7 | (7.2) | 0.641 | 54.8 | (6.7) |
| Mental component | 46.2 | (12.0) | 0.005 | 47.4 | (11.4) | 0.041 | 51.8 | (8.4) |

IQ = intelligence quotient, SD = standard deviation, VLBW = very low birth weight.

^a^Without cerebral palsy and/or estimated intelligence quotient <2SD of the mean in the control group

^b^Compared with 72 controls due to missing IQ data for seven control participants.
