# Supplementary material for: Health-related quality of life from 20 to 32 years of age in very low birth weight individuals: a longitudinal study
Source: Health Qual Life Outcomes. 2022 Sep 14;20:136. doi: 10.1186/s12955-022-02044-3 (PMC9476299; doi:10.1186/s12955-022-02044-3)
Supplement: Supplementary file 4 — Additional file 4: Table S4 Estimated changes in health-related quality of life from 20 to 32 years in participants without disabilities [file 12955_2022_2044_MOESM4_ESM.docx]

**Table S4** Estimated changes in health-related quality of life from 20 to 32 years in participants without disabilities

|  | **VLBW without disabilities^a,b^**  (n=50) | | | | | | **Control^b^**  (n=86) | | | | | | p-value time*group |
| --- | --- | --- | --- | --- | --- | --- | --- | --- | --- | --- | --- | --- | --- |
|  | 20 to 23 years | | 23 to 28 years | | 28 to 32 years | | 20 to 23 years | | 23 to 28 years | | 28 to 32 years | |  |
|  | B | (95% CI) | B | (95% CI) | B | (95% CI) | B | (95% CI) | B | (95% CI) | B | (95% CI) |  |
| Domains |  | | | | | |  | | | | | |  |
| Physical functioning | -3.6 | (-6.3 to -1.8) | 2.8 | (0.3 to 6.1) | -4.2 | (-8.7 to 1.5) | 0.4 | (-2.0 to 3.3) | 1.0 | (-1.6 to 3.3) | -1.3 | (-2.8 to -0.4) | 0.131 |
| Role-physical | -4.0 | (-13.4 to 2.4) | -1.2 | (-13.2 to 13.5) | -4.6 | (-15.9 to 8.6) | 4.5 | (-4.7 to 13.6) | -5.8 | (-11.5 to -0.7) | 2.2 | (-3.4 to 8.0) | 0.375 |
| Bodily pain | -6.6 | (-17.1 to 4.7) | 3.1 | (-5.8 to 12.9) | -1.8 | (-11.0 to 8.4) | 0.7 | (-4.4 to 6.1) | -1.1 | (-3.6 to 6.1) | -4.1 | (-8.3 to 0.1) | 0.517 |
| General health | -12.8 | (-18.3 to -7.5) | 6.9 | (0.6 to 13.4) | -9.9 | (-16.5 to -3.8) | -4.9 | (-9.9 to -0.2) | 10.1 | (4.6 to 16.6) | -6.8 | (-9.9 to -5.6) | 0.001 |
| Vitality | -5.2 | (-10.4 to -0.2) | 1.4 | (-3.6 to 6.4) | 1.1 | (-4.2 to 7.5) | -1.7 | (-7.3 to 3.5) | 2.2 | (-3.0 to 8.4) | -1.6 | (-4.9 to 1.3) | 0.617 |
| Social functioning | -2.6 | (-8.1 to 2.5) | -2.2 | (-8.4 to 3.4) | -2.2 | (-9.4 to 5.7) | 1.6 | (-1.6 to 4.6) | 0.7 | (-3.7 to 5.0) | -3.8 | (-7.1 to -0.1) | 0.323 |
| Role-emotional | -8.1 | (-16.8 to 0.1) | -3.9 | (-15.6 to 6.9) | -4.6 | (-14.7 to 6.2) | 6.0 | (-1.1 to 12.8) | -2.8 | (-9.9 to 4.6) | -0.4 | (-4.2 to 3.1) | 0.002 |
| Mental health | -5.4 | (-9.6 to -1.4) | 1.7 | (-3.2 to 7.0) | 2.5 | (-2.4 to 8.4) | -0.7 | (-4.6 to 2.6) | 3.1 | (-1.5 to 8.1) | -1.4 | (-3.8 to 0.8) | 0.189 |
| Component summaries |  |  |  |  |  |  |  |  |  |  |  |  |  |
| Physical component | -2.5 | (-5.0 to -0.4) | 1.9 | (-0.3 to 4.5) | -2.6 | (-5.6 to -0.9) | 0.1 | (-1.6 to 1.7) | 0.4 | (-1.3 to 2.0) | -0.8 | (-2.0 to 0.2) | 0.184 |
| Mental component | -2.6 | (-5.3 to 0.1) | -0.7 | (-3.8 to 2.7) | 0.6 | (-2.4 to 4.0) | 0.1 | (-2.3 to 2.1) | 0.8 | (-1.7 to 3.7) | -0.8 | (-2.3 to 0.5) | 0.082 |

CI = confidence interval, SD = standard deviation, VLBW = very low birth weight.

^a^Without cerebral palsy and/or estimated intelligence quotient <2SD of the mean in the control group.

^b^Missing estimated intelligence quotient for four VLBW and 14 control participants.
